# The Future of Musical Emotions

**DOI:** 10.3389/fpsyg.2017.00988

**Published:** 2017-06-19

**Authors:** Dylan van der Schyff, Andrea Schiavio

**Affiliations:** ^1^Faculty of Education, Simon Fraser UniversityVancouver, BC, Canada; ^2^Institute for Music Education, University of Music and Performing Arts GrazGraz, Austria; ^3^Music Mind Machine in Sheffield, Department of Music, The University of SheffieldSheffield, United Kingdom

**Keywords:** musical emotions, basic emotions, affective science, dynamical systems theory, music cognition

## Introduction

Basic Emotion Theory proper (BET) has only recently begun to make an appearance in musical research (see Juslin, 1997, 2013a,b). However, much theory and research in music psychology has been driven by a more general assumption that musical emotions should be investigated in terms of discrete *ad-hoc*^1^ categories associated with the ways specific neural mechanisms respond to musical stimuli (see Schiavio et al., 2016). This has been problematized, however, by studies that show that the physiological changes associated with musical emotions do not always align clearly with those exhibited in association with everyday emotion categories (Krumhansl, 1997, p. 351; Scherer and Zentner, 2001). Such concerns have led some scholars to posit that musical emotions may be somehow different (or perhaps “impoverished”) versions of real emotions (Sloboda, 2000). In response to this, other researchers (e.g., Scherer and Coutinho, 2014) have developed models that do away with the notion of basic emotions altogether, preferring instead to describe emotional reactions to music in terms of complex information processing components that combine in various ways to produce relevant outputs (see also Huron, 2006). Still others (Krueger, 2013; van der Schyff, 2013; Schiavio et al., 2016; see also Koelsch, 2013) have suggested that reducing musical experience to a stimulus-response framework—where emotions are thought to be caused in listeners by pre-given stimuli in the environment—may play down the active and creative role living embodied agents play in musical experience.

With this in mind, we offer below a brief critique of BET, suggesting that it may not in fact provide the best way forward for research in musical emotions. We then outline an alternative perspective, drawing on research that employs dynamical systems theory (DST) (Lewis and Granic, 2000; Colombetti, 2014). To conclude, we offer some preliminary suggestions for how this approach might be applied in musical contexts.

Before we begin, it should be noted that musical research that draws on the idea of basic emotions has indeed produced important insights^2^. Such studies are carried out in controlled settings that adhere to high scientific research standards—they offer important sources of data that will have to be taken into consideration by any alternative theoretical orientation. To be clear, then, our aim is not to debunk or discredit the work of researchers endorsing BET. Rather, our goal is simply to outline another perspective that could make important contributions to the dialogue^3^.

## Basic emotions

In folk psychology, the notion of “basic emotions” involves the assumption that emotions come in a group of pre-defined categories such as happy, sad, angry, and so on. This idea has been formalized by BET proper, which claims the existence of a small group of “discrete” basic emotions that evolved in response to the challenges our ancestors faced in their everyday life (Ekman, 1980). It is argued that each basic emotion is defined by a dedicated (innate and distinct) neural network, which controls the “affect programs” that give rise to them (Tomkins, 1962, 1963; see also Colombetti, 2014). Here the term “affect programs” refers to the mechanisms that produce “the patterns for these complex organized responses, and which when set off directs their occurrence” (Ekman, 1980 p., 82). Such programs and the responses they trigger (i.e., basic emotions) are thought to be culturally universal and are thus understood to function as “auto appraisals” selected by evolution (Ekman and Friesen, 1971, 1986; Ekman, 1999, 2003). It has also been suggested that because of the supposed categorical fixity of these basic emotions, they might form the “building blocks” from which more complex emotions emerge (Prinz, 2004a,b).

Some critics have raised the question of how, precisely, alleged basic emotions relate to non-basic ones (Ortony and Turner, 1990). And there is an ongoing debate between those who argue that basic and non-basic emotions are continuous (Plutchik, 2001; Prinz, 2004a,b; Clark, 2010), and those who insist that the two involve distinct processes and should thus be studied separately (Damasio, 1994; LeDoux, 1996). Other authors question BET's lack of context sensitivity (Russell, 2003; Barrett, 2006): emotions can be expressed and experienced in various ways and their meanings shift depending on the situation. Attention has also been drawn to the BET methodology, which often employs a forced choice approach (generally between facial expressions and a given scenario). Indeed, research has shown that quite different responses may be given when participants are not constrained by emotional labeling (Russell, 1994). It has also been pointed out that the close correlations that are expected to occur between specific brain areas, autonomic nervous system activity, and the putative affect programs associated with basic emotions are often less consistent than hypothesized (Barrett, 2006). While advocates of BET have responded to such criticisms in various ways (e.g., Ekman and Cordaro, 2011), another more general problem remains concerning the rather arbitrary way basic emotion categories were originally introduced^4^. To be clear, all of this does not negate the observation that emotional episodes involve recurring psycho-physical patterns of behavior that are indeed nameable, and that may bear striking similarities across individuals and groups. Rather, it suggests that we need to develop new approaches for studying such phenomena.

## Emotions as dynamic and emergent

Research based on DST suggests that emotional episodes may not be reducible to pre-given response mechanisms in the brain, but rather emerge through developmental processes spanning an integrated body-brain-environment network (Freeman, 2000; Lewis, 2000, 2005). Colombetti (2014, p. 57–82) suggests three mutually informing levels of inquiry that may help us better understand how this is so. The first of these involves the development of the muscular linkages and coordinative structures associated with emotional expression (e.g., facial, vocal, limb, and hand movement). And indeed, research shows that the appearance of recurrent and meaningful patterns of expressive behavior in infants is not best understood wholly in terms of predetermined (i.e., genetic) developmental programs, but rather as emergent properties of the dynamic interaction of a range of environmental and bodily factors (Fogel and Thelen, 1987; Camras and Witherington, 2005). The second level draws on existing work in affective neuroscience that highlights the plasticity and self-organizing nature of neural structures (see Freeman, 1999, 2000). Here, emotional episodes are explored as patterns of convergence (basins of attraction) in the neural trajectories of an agent that may both stabilize and transform due to contingent shifts in the global constraints of the brain-body-world system. The third level focusses on environmental concerns, exploring how agents enact meaningful emotional engagements (i.e., recurrent patterns of relational behavior) with the things and people they interact with, and the situations they live through (Laible and Thompson, 2000; Hsu and Fogel, 2003). From this perspective, emotional episodes are not limited to the skull or the skin of the organism. There is, rather, a strong sense in which they “extend” (Krueger, 2014; Slaby, 2014; Krueger and Szanto, 2016) into environments where agents co-enact meaningful worlds through forms of participatory sense-making (such as music; see Schiavio and De Jaegher, 2017).

In brief, the DST approach explores the *active* and *self-organizing* nature of emotional experience as it develops across bodily, neural, and ecological dimensions, suggesting that while emotional episodes can indeed be unique to an individual organism, they may also emerge in ways that are shared between agents with similar biological/social needs and histories of interaction with the environment.

## Exploring the dynamics of musical emotions

It should be noted that work in music cognition has already begun to develop DST approaches. For example, researchers (e.g., Large and Jones, 1999) have investigated how the neural dynamics associated with musical experience actively resonate and self-organize with those of the body and the environment in a recursive or “circular” way. However, such “dynamic attending” to music has been explored mostly in the context of rhythmic entrainment and pitch perception (e.g., Large et al., 2016; see also McGrath and Kelly, 1986). We suggest that future research could develop the emotional implications of this more fully. Indeed, this would contribute to a larger body of inter-disciplinary work that explores the creative, social, embodied, ecological, and developmental aspects of musical experience (DeNora, 2000; Leman, 2007; van der Schyff, 2015).

For example, it has been shown that even in contexts that appear to be “passive” (e.g., listening in a concert hall) people play active roles in shaping their engagements with musical environments^5^ (Clarke, 2005; Krueger, 2014)—e.g., by developing “metaphorical,” cross-modal, and “narrative” relationships between various temporal, spatial, textural, bodily, social, ecological, and affective dimensions (see Johnson, 2007). Here, DST could be useful for exploring how such experiences are enacted, especially when integrated with phenomenological descriptions^6^.

This orientation could also be developed in contexts involving the production of music (performance, rehearsal, instrumental practice, and so on). Indeed, because musical performance clearly involves the integration of the three levels of description discussed above (bodily/neural/environmental), DST might be used to investigate and describe situations where two or more individuals participate in realizing the same musical event. This could include the use of electrophysiological measurements in association with techniques such as functional near-infrared spectroscopy (fNIRS), which are increasingly adopted in the context of social cognition (Osaka et al., 2015; Pu et al., 2016).

As fNIRS allows for the measurement of hemodynamic activities in participatory settings it could be particularly useful for exploring music-related activities in terms of interpersonal emotional dynamics. These could be analyzed in conjunction with audio and video recordings (to help capture sonic, bodily, and other environmental aspects); as well as through interviews with the participants to better understand how various forms of musically adaptive behavior might initiate (and be driven by) emotional episodes that are both recurrent and new, and how such episodes are experienced by the participants (e.g., as shared and/or as personal). Examples of similar data collection methods come from work by Walton et al. (2014, 2015) who have used DST to study perceptions of creativity in interacting musical improvisers (see also Borgo, 2005; Laroche and Kaddouch, 2015)^7^.

In all, we suggest that a DST approach may better capture the (inter)active ways people enact emotional relationships with music, and how such experiences develop diachronically. That is, it may help us better understand how musical emotions emerge and transform developmentally in the context of people's lives—where stable or recurrent patterns of behavior may come to be experienced in similar ways between embodied agents, and thus be subject to “loose” labeling without being wholly pre-determined, fixed, or simply reducible to discrete categories. Likewise, it may also provide insights into the more idiosyncratic ways we engage emotionally with music—e.g., the conscious ways individuals seek out new approaches to music making as a means of creative expression, or as a way of enacting meaningful but highly personal embodied-emotional relationships (aesthetic, social, therapeutic) with the environments they inhabit (DeNora, 2000).

## Conclusion

While BET-based approaches will continue to provide many useful insights in more controlled environments, we suggest that the DST perspective may better capture the manifold ways emotional engagement with music unfolds in the complex, embodied, and socially interactive contexts that characterize lived experience. Because this approach sees emotions as emergent and enacted, and not first in terms of pre-given categories or “affect programs,” it arguably sidesteps the problematic issue introduced above regarding whether musical emotions correspond with “basic,” “real,” or “everyday” emotions. In line with this, it would be very interesting to see how the results of musical research that assume a basic emotion approach might be reinterpreted from a DST perspective. Additionally, DST might also shed light on important aspects of musical development in early childhood^8^. Lastly, because DST-based research sees emotionality as inextricable from our embodied and social existence, it could have more general implications for areas such as music education and therapy—perhaps providing ways for teachers, students, therapists, and patients to better understand and discuss their engagements with music and its meaning for their lives.

## Author contributions

The authors have made equal contribution to this paper and are thus both considered as first authors.

### Conflict of interest statement

The authors declare that the research was conducted in the absence of any commercial or financial relationships that could be construed as a potential conflict of interest.

## Footnotes

^1^Indeed, these categories tend to vary from study to study. Some have focused on more “basic” emotions (e.g., joy and sadness), while others introduce categories that might be better described as “moods” (e.g., tenderness or nostalgia). Recent approaches have developed more nuanced models that introduce complex “aesthetic” categories such as wonder and awe. For useful overviews see Juslin and Laukka (2004), Juslin and Västfjäll (2008), Eerola and Vuoskoski (2013), Trost et al. (2013).

^2^This includes the work of Juslin (1997, 2013a,b) who argues that BET proper should be adopted more widely in musical emotion studies. In line with this, recent research has explored (among other things) the relationship between perceived emotions and physiological changes (Lundqvist et al., 2008), as well as how emotional categories may be recognized consistently across listeners (Fritz et al., 2009).

^3^Indeed, comparisons of research results between competing programs may lead to important new discoveries and avenues of investigation.

^4^This refers to the work of Tomkins (1962, 1963), who posited the existence of nine primary affect categories (1962, p. 111–112). While no sustained justification was given by Tomkins for why these categories were chosen, subsets of them have nevertheless been assumed to comprise the basic emotional responses that guide our affective engagements with the world. For a full discussion see Colombetti (2014), p. 36–40.

^5^Here one might also consider the ways people use personal music listening devices to regulate their emotions (Skånland, 2013) in everyday life and to transform or “aestheticize” the environments they live though (Bull, 2000, 2007). This could offer yet another avenue to explore from a dynamical perspective.

^6^Here the recently developed “4E” model (*embodied, embedded, enactive*, and *extended*) associated with enactive cognitive science could provide a general framework for guiding the phenomenological aspects of such research (for a brief overview see van der Schyff, 2017; for a range of more detailed perspectives see Menary, 2010).

^7^On a related note, readers may also be interested to consider a study by Salice et al. (2017) that examines the enmeshed embodied, social, affective, and musical dynamics of a string quartet to explore the phenomenon of “plural self-awareness.”

^8^E.g., the emergence of the (proto)musical forms of embodied-affective communication that characterize the interactions between infants and primary caregivers (see Trevarthen, 2002); as well as the independent and collaborative exploratory musical activities of young children.
